# Modeling Focused Ultrasound Exposure for the Optimal Control of Thermal Dose Distribution

**DOI:** 10.1100/2012/252741

**Published:** 2012-04-19

**Authors:** E. Sassaroli, K. C. P. Li, B. E. O'Neill

**Affiliations:** Department of Radiology, The Methodist Hospital Research Institute, Weill Medical College of Cornell University, 6565 Fannin Street, MS B5-011, Houston, TX 77030, USA

## Abstract

Preclinical studies indicate that focused ultrasound at exposure conditions close to the threshold for thermal damage can increase drug delivery at the focal region. Although these results are promising, the optimal control of temperature still remains a challenge. To address this issue, computer-simulated ultrasound treatments have been performed. When the treatments are delivered without taking into account the cooling effect exerted by the blood flow, the resulting thermal dose is highly variable with regions of thermal damage, regions of underdosage close to the vessels, and areas in between these two extremes. When the power deposition is adjusted so that the peak thermal dose remains close to the threshold for thermal damage, the thermal dose is more uniformly distributed but under-dosage is still visible around the thermally significant vessels. The results of these simulations suggest that, for focused ultrasound, as for other delivery methods, the only way to control temperature is to adjust the average energy deposition to compensate for the presence of thermally significant vessels in the target area. By doing this, we have shown that it is possible to reduce the temperature heterogeneity observed in focused ultrasound thermal treatments.

## 1. Introduction

Local temperature elevation (hyperthermia) has been investigated for the treatment of many kinds of cancer for several years [1–5]. Early clinical trials that took place in the 1990s pointed out challenges associated with thermal therapies such as difficulty in reaching most tumor sites. For the limited number of sites that were heatable, dosimetric studies indicated that the temperature distributions reached were highly inhomogeneous and that it was almost impossible to obtain the protocol temperature goals [6, 7]. Accordingly, it is generally believed that the most beneficial contribution of hyperthermia for cancer treatment is based on enhancing the effectiveness of other treatment modalities such as radiotherapy or chemotherapy [8]. As a matter of fact, several clinical studies have shown a statistically significant tumor control when hyperthermia is combined with radiotherapy, chemotherapy, or both [9, 10]. Recent studies have indicated that this benefit may arise in part from heat-induced sensitization of cancer stem or tumor initiating cells to radiation or chemotherapy [11].

In the field of ultrasound hyperthermia, to overcome the unsatisfactory results caused by technical and temperature control-problems during the clinical use of the first-generation scanned-focused ultrasound systems [12, 13], a new generation of focused ultrasound systems have been built for MRI guidance and thermometry that are characterized by long treatment times and small heated regions [14, 15]. These systems are now being tested extensively for variety of applications besides tumor ablation [16, 17], including local drug delivery [18], control of gene therapy [19], and blood-brain-barrier disruption [20].

From the clinical side, MRI-guided-focused ultrasound has been approved by the Food and Drug Administration for the treatment of uterine fibroids [21, 22] and is in clinical trials/investigation for ablation of brain tumors [23], breast tumors [24, 25], liver tumors [26] and as a palliative of pain caused by bone metastases [27].

All these possible clinical applications of MRI-guided-focused ultrasound would greatly profit from the development of ultrasound treatment planning [8]. This planning should accurately model (a) power deposition and acoustic energy absorption by the various tissues exposed to focused ultrasound and (b) the resulting temperature and thermal dose distribution in the treated area. Therefore, it should be able to deal with the complex relation between focused ultrasound system, perfusion, discrete vasculature, and anatomy. This is an extremely complex task but it is essential for optimizing the quality of focused ultrasound treatments.

We are particularly interested in the development of techniques involving MRI-guided-focused ultrasound for increasing local drug delivery. We have shown an increase in tissue permeability when the temperature is kept just below the threshold for thermal damage [18]. A region of focal thermal ablation surrounded by an area of increased tissue permeability with no apparent damage was observed in these studies. The optimal control of temperature in such a situation still remains a challenge. Our previous studies have addressed the difficulties inherent in producing uniformly low thermal doses when treating large regions with a system having limited electronic steering [28] and in capping thermal dose with temperature measurements of limited spatial and temporal resolution [29]. Neither of these studies considered the potential impact of vasculature on the temperature achieved. This issue is addressed in the present investigation. More specifically we have simulated temperature increase and thermal dose deposition produced by focused ultrasound treatments of a homogeneous block of muscle-like tissue with either (i) one vessel, (ii) one-vessel pair, or (iii) multiple vessel pairs.

Although several investigations (see e.g., [30] for a complete review) have considered the issue of blood flow in thermal treatments, our focus is to explicitly simulate different ultrasound delivery methods and to calculate the thermal dose associated to each treatment when thermally significant blood vessel pairs are taken into account. These calculations provide clear insights into the problems that need to be solved for the optimal control of thermal dose during focused ultrasound treatments.

## 2. Numerical Model

In our computer simulations, we have employed the bioheat transfer model developed by Lagendijk and coworkers [31–34]. In their model, the bioheat transfer equation is separated into an equation valid for the tissue domain and an equation valid for the vessel domain. These equations are coupled through their common boundary (the blood vessel walls). The tissue domain is described by the enhanced conductivity equation

(1) $$\rho_{t}c_{pt}\frac{\partial T}{\partial t}=\nabla \cdot \left(k_{\text{eff}}\nabla T\right)+P_{\text{FUS}},$$

where *T* is the temperature, *ρ*_*t*_ is the tissue mass density, *c*_*pt*_ is the tissue specific heat, *P*_FUS_ is power per unit volume produced by the external heating source (focused ultrasound), and *k*_eff_ is an effective conductivity which takes into account the presence of the smallest vessels that cannot be possibly modeled individually. In the tissue domain, we have solved (1) with the following parameters: *ρ*_*t*_  = 1000 kg/m^3^, *c*_*pt*_  = 4000 JKg^−1 ^K^−1^, and *k*_eff_ = 1.8 W m^−1^ K^−1^ [35].

In the vessel domain, the following heat transfer equation is solved:

(2) $$\rho_{b}c_{pb}\left(\frac{\partial T}{\partial t}+v\cdot \nabla T\right)=\nabla \cdot \left(k_{b}\nabla T\right)+P_{\text{FUS}},$$

where *ρ*_*b*_ is the blood mass density, *c*_*bt*_ is the blood specific heat, *k*_*b*_ is the blood heat conductivity, and **v** is the convective blood velocity. Since we are considering straight blood vessels, (2) can be simplified as

(3) $$\rho_{b}c_{pb}\frac{\partial T}{\partial t}=\nabla \cdot \left(k_{b}\nabla T\right)-\rho c_{pb}w\frac{\partial T}{\partial z}+P_{\text{FUS}},$$

where we have assumed *ρ*_*b*_ = 1060 kg/m^3^, *c*_*pb*_ = 3840 JKg^−1^ K^−1^  and *k*_*b*_ = 0.6 W m^−1^ K^−1^. The axial blood velocity *w* (*z*-direction in our computational domain) is given by

(4) $$w=2V_{m}\left(1-\frac{r^{2}}{R^{2}}\right)$$

with *V*_*m*_ mean blood velocity, *R* vessel radius, and *r* radial coordinate. The values for *V*_*m*_ and *R* have been taken from the literature [36] and represent generic values for representative vessels of a given size.

The power density distribution *P*_FUS_ produced by the focused ultrasound field is modeled as described in [16]

(5) $$P_{\text{FUS}}=P_{0}A\left(x,R_{x}\right)A\left(y,R_{y}\right)A\left(z,R_{z}\right)$$

with *A* an expression of the type

(6) $$A\left(u,R_{u}\right)=\exp\left(-\ln\left(2\right)\frac{u^{2}}{R_{u}^{2}}\right),$$

where the coefficients *R*_*u*_ are obtained by the half-power width and height and *P*_0_ is the power density (Wcm^−3^). These values are usually estimated experimentally.

Equations (1) and (3)–(6) have been solved using the finite element method on a geometry consisting of a block of muscle-like tissue containing either (i) one blood vessel, (ii) an artery-vein pair system, or (iii) a multiple set of artery-vein pairs. The finite element method has been implemented through COMSOL Multiphysics 3.5a and Matlab R2009b and subject to the following boundary conditions. For the tissue domain, the boundaries are related to the core temperature (37°C) by a fixed heat transfer coefficient *h* simulating a few centimeters of tissue with *h* = *k*_eff_/*d* and *d* the tissue thickness. The temperature at the vessel inlet is assumed to be 37°C and a convective heat flux is assumed at the outlet of the vessels. The initial temperature at *t* = 0 is assumed to be 37°C.

The overall performance of the ultrasound treatments is evaluated by calculating the thermal dose for the midplane of the computational domain. The thermal dose is calculated using the empirical relationship introduced by Sapareto and Dewey [37]

(7) $$TD\left(t\right)=\overset{t}{\underset{0}{\int }}{ℛ}^{\left(T_{\text{ref}}-T(t^{\prime })\right)}dt^{\prime },$$

where *T*_ref_ is the reference temperature. A temperature of 43°C was chosen as reference temperature since it is commonly used in reporting experimental thermal data. The value of *ℛ* (isodose constant) given by

(8) ℛ={0.25T(t)<43°C,0.5T(t)≥43°C

was chosen based on experimented data [37–39]. The boundary of an isothermal dose value of 240 min at the reference temperature of 43°C was selected to predict the size of the necrosed tissue volume in muscle [38, 40]. All thermal dose calculations are normalized to this standard in this study, with a thermal dose of one corresponding to the condition that will lead to 100% tissue necrosis.

## 3. Numerical Results

### 3.1. Single Vessel and Focused Ultrasound

To begin with, we have investigated the effect of vessel size and flow velocity on the temperature distribution during a focused ultrasound exposure of a single straight blood vessel.

**Figure 1:**
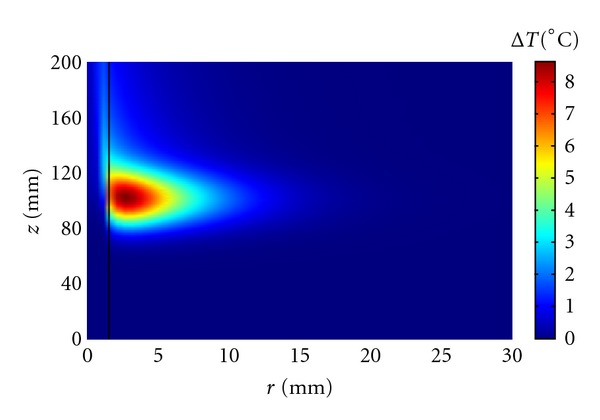
Axial symmetrical temperature increase reached at the end of a 60 s insonation period in a large artery (*R* = 1.5 mm, *L* = 200 mm, *V*_*m*_ = 13 cm/s). The ultrasound beam is directed downward and the blood flow is moving upward.

Initially, we considered a large straight artery with radius *R* = 1.5 mm, length *L* = 200 mm, and mean flow velocity *V*_*m*_ = 13 cm/s [36] running along the axial direction of the transducer with the ultrasound focus at the blood vessel center and the blood flow opposite to the ultrasound beam propagation (Figure 1). In this geometry, the model can be solved using axial symmetry, which simplifies the simulations.

This is the worst case scenario since the blood flow takes away the heat generated inside the vessel. For the purpose of illustration, we assumed the following ultrasound parameters: focus half-power width 4 mm, height 30 mm, and peak power density *I*_0_ = 15 W/cm^3^. These values are similar to the ones reported in [41]. As expected, Figure 1 shows that even after one minute of insonation, the blood temperature increase remains low and the heat propagation is not symmetrical because of the blood flow presence.

**Figure 2:**
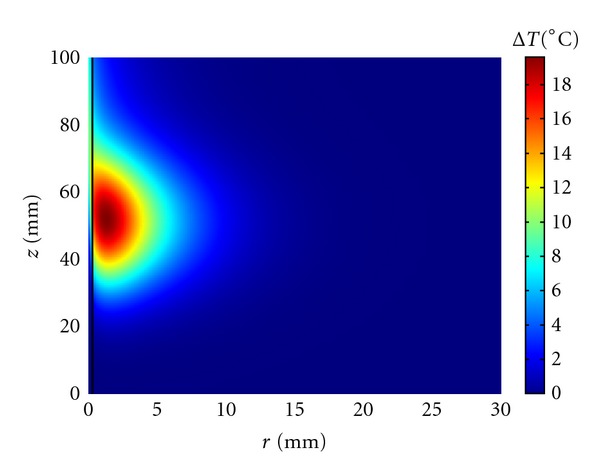
Axial symmetrical temperature increase reached at the end of a 60 s insonation period in a primary artery (*R* = 0.5 mm, *L* = 100 mm, *V*_*m*_ = 8 cm/s). The ultrasound beam is directed downward and the blood flow is moving upward.

**Figure 3:**
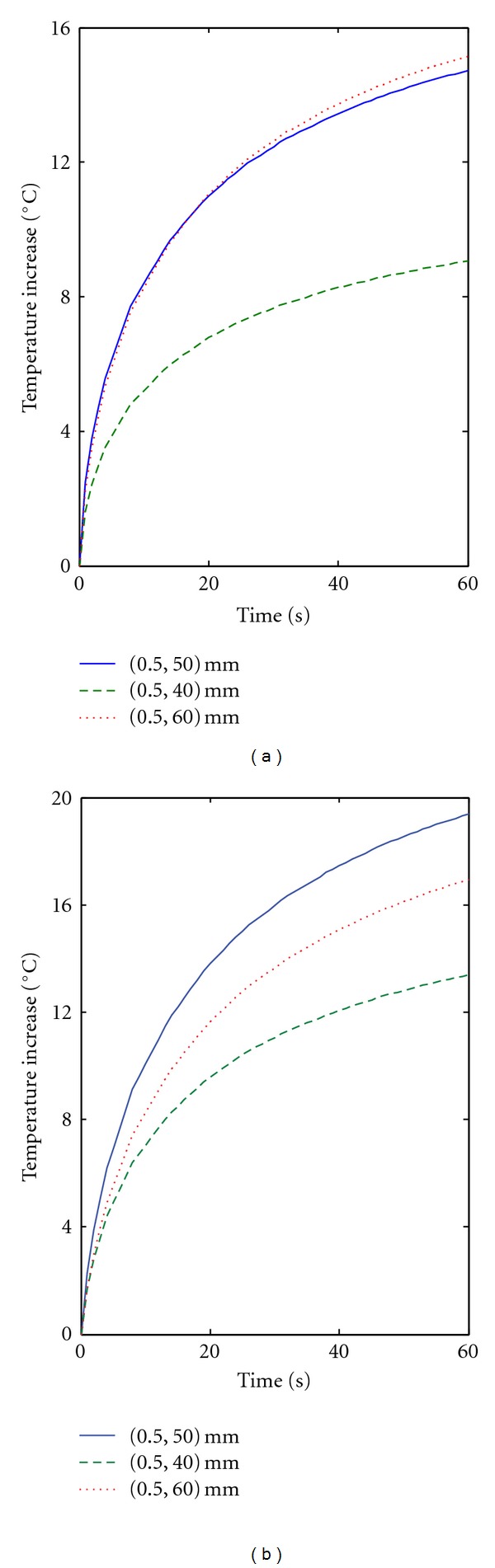
Temperature increase as a function of time for points (a) on the vessel wall and (b) 1 mm away the vessel wall. The vessel parameters and ultrasound parameters are as in Figure 2 (primary artery). For each plot, the points are on the focal plane (*z* = *L*/2 = 50 mm), 10 mm below, and 10 mm above it.

The same calculations have been repeated for a main branch of a large artery (primary artery) with radius *R* = 0.5 mm, length *L* = 100 mm, and mean velocity *V*_*m*_ = 8 cm/s [36]. As may be seen in Figure 2, the heating in the focal region after one minute of insonation is much higher than the one shown in Figure 1 for the largest vessel. In Figure 3, we plotted the temperature increase as a function of time for points which are on the vessel wall and 1 mm away from the vessel. These points are at the focal plane (*z* = *L*/2 = 50 mm), 10 mm below (far field), and 10 mm above it (near field). Sustained heating is obtained at the vessel wall in the focal plane and 10 mm above the focal plane, at the risk, however, of having possible thermal damage 1 mm from the vessel wall.

**Figure 4:**
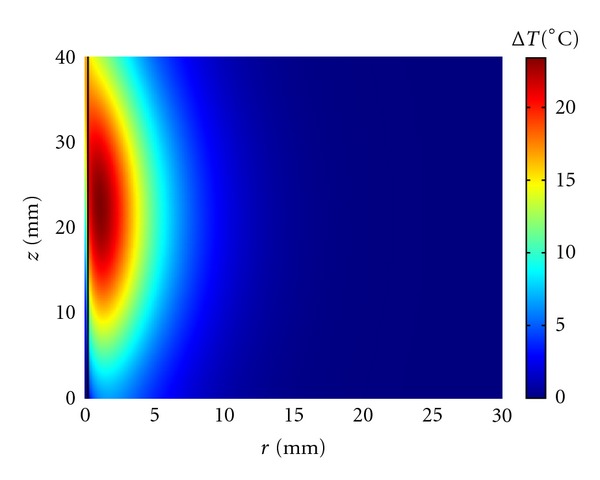
Axial symmetrical temperature increase reached at the end of a 60 s insonation period in a secondary artery (*R* = 0.3 mm, *L* = 40 mm, *V*_*m*_ = 8 cm/s). The ultrasound beam is directed downward and the blood flow is moving upward.

If we consider a secondary branch with typical values *R* = 0.3 mm, *L* = 40 mm, and *V*_*m*_ = 8 cm/s [36], the cooling effect exerted by blood flow is not as important as for the previous two cases. After one-minute insonation at the same exposure conditions as in Figures 1 and 2, the blood temperature increase is significant at least at the focal plane and above it as may be seen in Figure 4.

**Figure 5:**
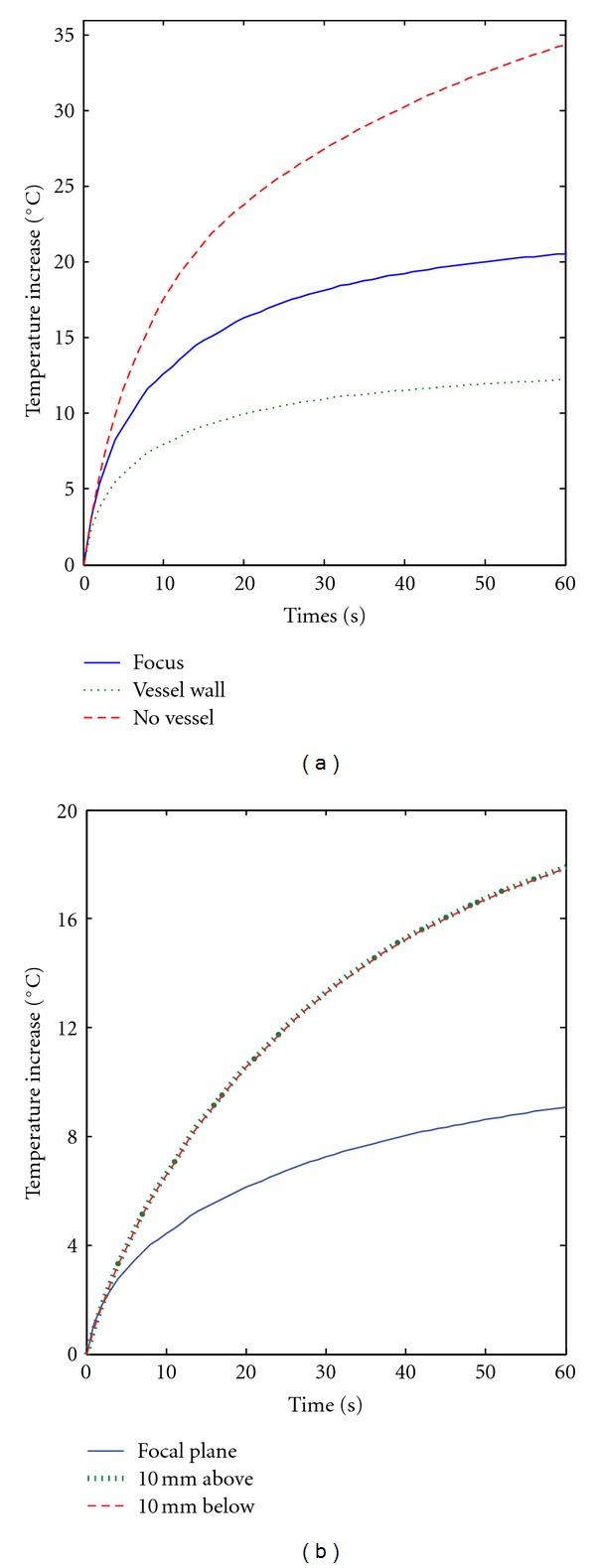
Temperature increase as a function of time for points 1 mm away from the vessel wall. The vessel and ultrasound parameters are as in Figure 1 (large artery), except that in (a) the position of the ultrasound focus is 1 mm away from the vessel wall and in (b) the vessel makes an angle of 90°C with the direction of propagation of the ultrasound field. The plots have been obtained through a full 3D simulation.

When the ultrasound focus is not at the vessel center and/or the vessel makes an angle different from zero with the axial direction of the transducer, the axial symmetry is lost and a full 3D simulation has to be preformed. When the focus is not inside the vessel, temperatures favorable to thermal ablation can be easily reached. These ideas are illustrated in Figure 5. The plots in this figure have been obtained assuming the large artery to be at the center of a simulation domain having dimensions 60 × 60 × 40 mm^3^. When the focus is 1 mm from the vessel wall, thermal ablation conditions are easily reached at the focal plane even for the largest vessel as may be seen in Figure 5(a), where the temperature increase at the focus has been plotted as a function of time for a 60 s insonation period. For comparison, the temperature at the focus without the vessel and the temperature on the vessel wall at the focal plane have been also plotted. In Figure 5(a), the parameters are the same as for Figure 1 except for the position of the focus that is 1 mm away from the vessel wall rather than at the vessel center.

Figure 5(b) shows the temperature increase as a function of time when the vessel makes an angle of 90° with the direction of propagation of the ultrasound beam. All the other parameters are the same as in Figure 1. The peak-temperature increase at the end of the insonation period is just a bit higher than the one with the vessel parallel to the *z* direction; however planes above and below the focal planes have a much higher temperature. A similar result is obtained, for example, when the vessel makes an angle of 45° with the *z* axis, that is, thermal ablation conditions are present above and below the focal plane and much smaller temperatures are seen in the focal plane (data not shown).

### 3.2. Artery-Vein Vessel Pair and Focused Ultrasound

**Figure 6:**
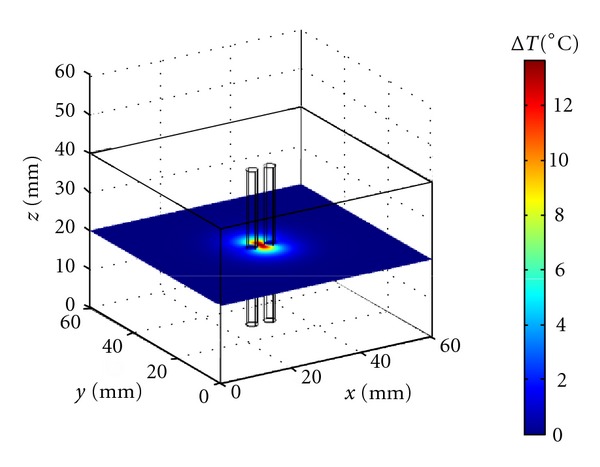
Temperature increase reached at the end of a 60 s insonation period at the focal plane for an artery-vein vessel pair and nearby tissue. The vessel and ultrasound parameters are the same as in Figure 1 and the ultrasound focus is at the center of the computational domain between the two vessels.

Up to the level of arterioles, venules and capillaries, and with the exception of the superficial venous system, vessels run in counterflow pairs and therefore it is very important to consider artery-vein vessel pair systems. In this study, however, we will limit our discussion to the geometry illustrated in Figure 6. The two vessels in Figure 6 represent an artery and a vein in a counterflow situation with vessel parameters typical of large arteries: *R* = 1.5 mm and *V*_*m*_ = 13 cm/s. In Figure 6, the focus is at the center location of the model block and at distance of one mm from each of the vessels. The presence of the two vessels significantly restricts the propagation of heat along the *x* direction and affects the overall temperature distribution in the focal region. For example, after one minute of insonation the peak temperature increase at the focal plane is about 35% lower than with no vessels present in the model and 38% lower 1 cm above and below the focal plane.

When the focus is at one of the vessel centers, the peak temperature increase in the focal region is similar to the one obtained when one single vessel is present (Figure 1). When the focus is 1 mm away from only one of the vessel walls, the situation is similar to the one described in Figure 5(a).

Therefore, the presence of the pair introduces temperature inhomogeneity by cooling some areas in the focal region while leaving some others unaffected.

### 3.3. Multiple Vessel Pairs and Focused Ultrasound

We have investigated a multiple artery-vein system located in a homogeneous block of muscle-like tissue with dimensions 21 × 21 × 26 mm^3^. For the purpose of illustration, we have considered the vessel pairs to run in parallel along the axial direction of the transducer and having a radius of either 500 *μ*m (3 pairs) or 400 *μ*m (6 pairs). These vessels are thermally significant according to [42–44]. The distance between the artery and vein is 0.8 mm for the 500 *μ*m pair and 0.6 for the 400 *μ*m pair. The focus half-power width and height are assumed to be, respectively, 3.2 and 24 mm and the power density distribution is varied during the treatment. The thermal treatment of the multiple vessel system is simulated by an ultrasound focus that is stepped through the midplane of the computational domain using different delivery methods. The following delivery methods have been adopted: (i) the focus is stepped through the midplane of the computational domain in a random way to avoid thermal build-up, (ii) the focus is stepped in a sequential manner, or (iii) the focus is stepped through a spiral trajectory starting at the center of the computational domain and going outward. In all the three delivery methods, the number of insonations is 56 and the duration time for each insonation is 20 s followed by a 5 s cooling period for a total insonation time of 1395s, about 23 minutes.

**Figure 7:**
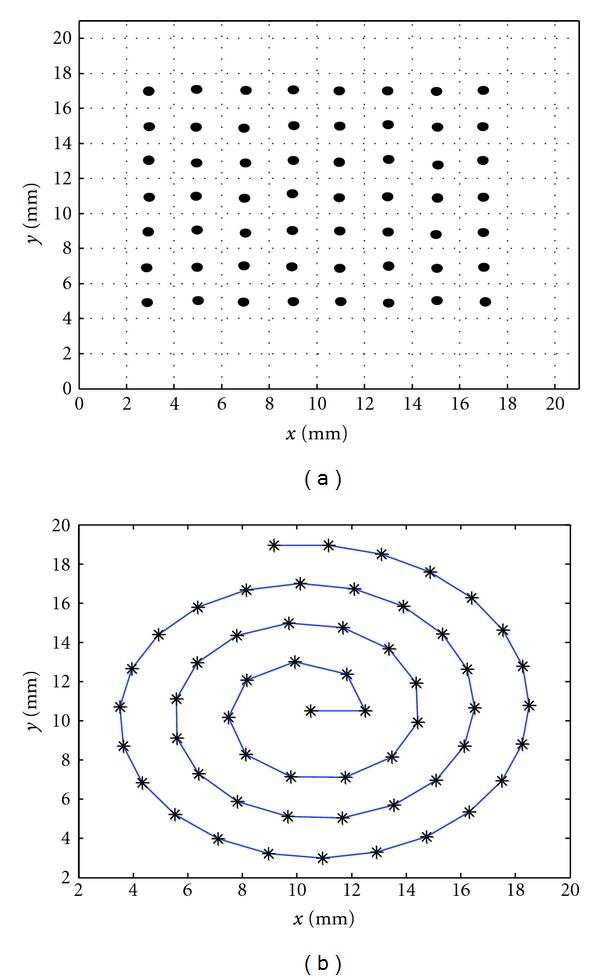
Points in the mid-plane of the computational domain through which the focus is stepped either in (a) a random way or sequential way, or (b) following a spiral trajectory.

Figure 7(a) shows the points through which the focus is stepped for cases (i) and (ii). The points are separated by a distance of 2 mm. For case (i), the focus is stepped in a random way though the 56 points with the condition that the distance between one insonation and the next is at least 8 mm. For case (ii) the focus is stepped in a sequential way starting from the top left square.

In the spiral trajectory case (iii), illustrated in Figure 7(b), the distance between two successive turns is 2 mm and also the distance between two successive insonation points is 2 mm.

**Figure 8:**
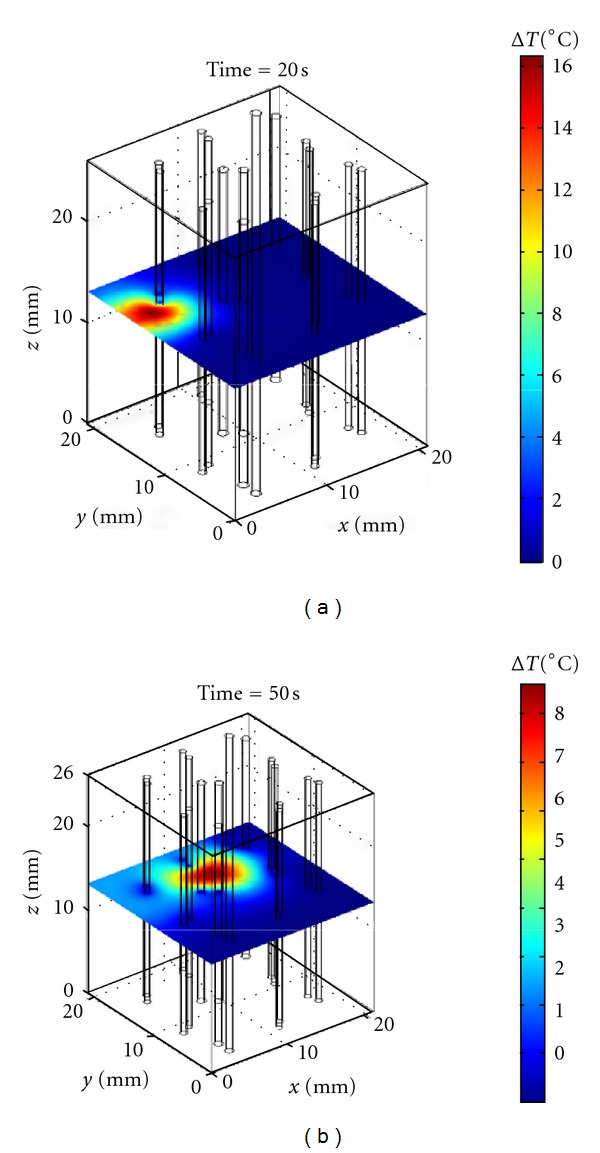
Temperature distribution reached in the midplane for the multiple artery-vein vessel pairs at the end of the first insonation period (a) and at the end of the second cooling period (b) for the random insonation treatment with peak power density of 17.5 W/cm^3^. The other parameters are illustrated in the text.

Figure 8 shows examples of temperature distribution in the midplane at the end of various insonation periods for the random insonation treatment. In Figure 8(a) the temperature distribution reached at the end of the first insonation period may be seen. The final peak temperature increase is about 16°C and the temperature distribution is distorted by the presence of a nearby vessel pair.  Figure 8(b) illustrates the temperature distribution reached at the end of the second cooling period. The final peak temperature increase is about 8°C and the heat propagation is influenced by the presence of the vessel pairs that represent a barrier to heat propagation.

**Figure 9:**
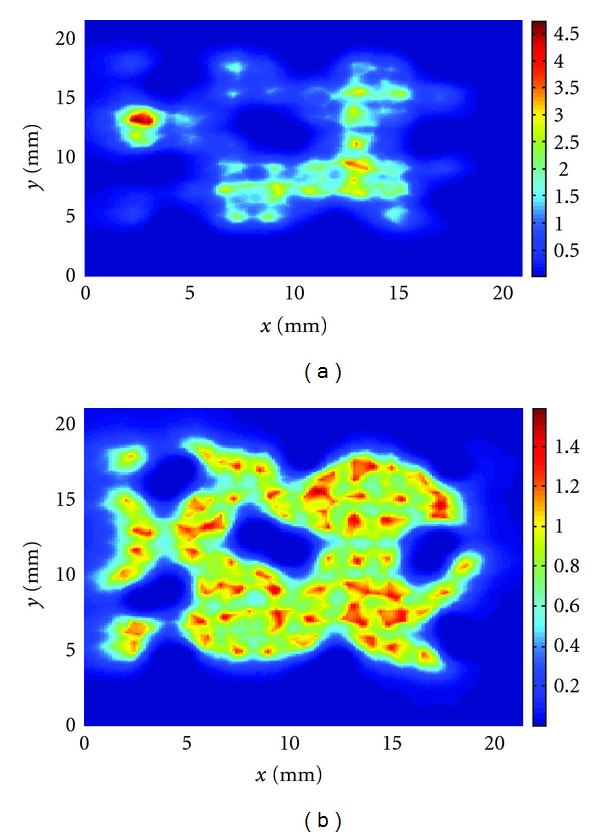
Total thermal dose accumulation in the midplane for the random insonation treatment (a) with fixed power and (b) with variable power.

**Figure 10:**
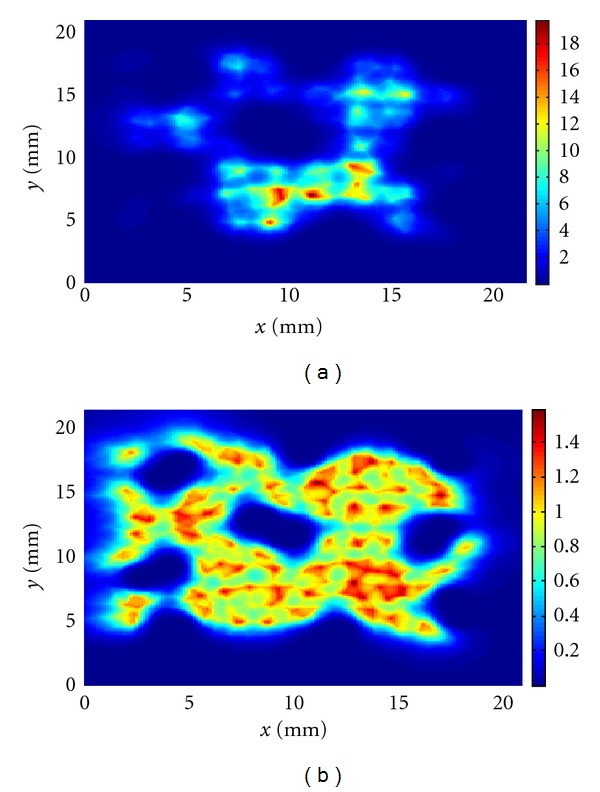
Total thermal dose accumulation in the midplane for the sequential insonation treatment (a) with fixed power and (b) with variable power.

**Figure 11:**
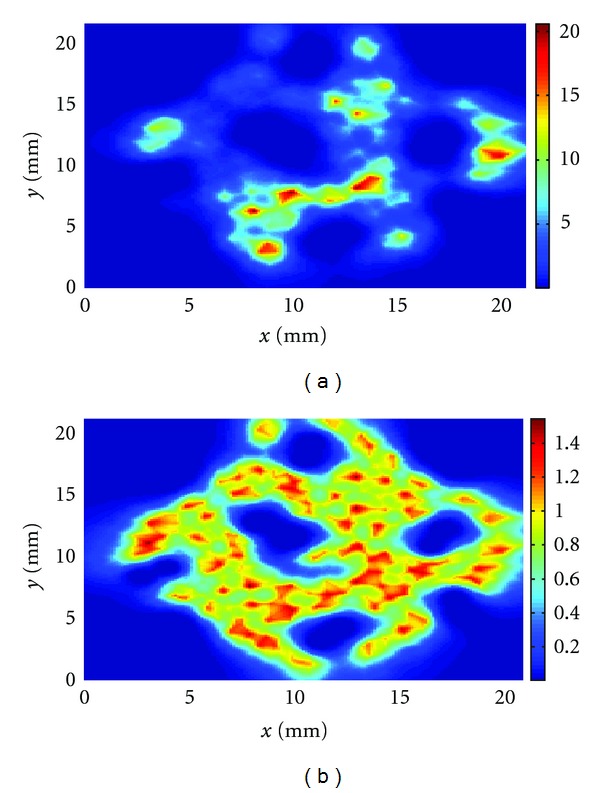
Total thermal dose accumulation in the midplane for the spiral insonation treatment (a) with fixed power and (b) with variable power.

We have then calculated the thermal dose in the midplane of the computational domain for cases (i), (ii), and (iii). At first, we have kept the power density distribution fixed during the treatments with a peak power of 16.5 W/cm^3^. In Figure 9(a), the thermal dose for the entire treatment for case (i) is shown. In this figure and in the following ones, the color bar gives the values of the thermal dose normalized with respect to the isothermal dose value of 240 min which is the threshold for tissue necrosis as described in Section 2. Figures 10(a) and 11(a) show the equivalent plots for cases (ii) and (iii). As may be seen in these plots, when the power is kept fixed during the treatments, the thermal dose deposition varies greatly from one delivery method to the other with the highest peak thermal dose deposition observed for the spiral treatment. In all the treatments, underdosage around the vessel pairs is clearly visible. The mean thermal doses are, respectively, for case (i) the mean is 1.07 and the standard deviation 0.65; case (ii) the mean is 3.2 and standard deviation 3.0; case (iii) the mean is 3.5 and standard deviation 3.2. These mean values have been obtained by averaging the thermal dose values above 0.3 in order to avoid the cooler regions inside the vessels.

In addition, we have manually changed the power distribution so that the peak thermal dose deposition remains in the range between 230 to 250 min for each insonation, with the aim of simulating a controlled feedback situation. The results of the simulations are given in Figures 9(b), 10(b), 11(b), respectively, for cases (i), (ii), and (iii). For case (i), the total peak thermal dose is 1.6 and the mean and standard deviation are, respectively, 0.8 and 0.2. For case (ii), the total peak thermal dose is 1.6 and the mean and the standard deviation are, respectively, 0.81 and 0.3. For case (iii), the total peak thermal dose is 1.5 and the mean and standard deviation are, respectively, 0.8 and 0.2. All these values have been calculated considering only thermal dose values above 0.3, as above. Hence, when the power is changed during the treatments, the thermal dose deposition is similar in all the three cases and the delivery method does not appear to be as important as in the examples with fixed power. Furthermore, the thermal dose is more uniform than when the power is kept fixed. However, underdosage is still visible around the vessel pairs.

## 4. Discussion

Besides factors such as absorbed power, thermal conductivity, and specific heat, blood flow is the main parameter which determines temperature distribution in tissues. In this study in order to describe the effect of blood vessels and blood flow on the temperature and thermal dose distribution reached during a focused ultrasound treatment, we have employed the bioheat transfer model developed by Lagendijk and co-workers. Models of the type proposed by Lagendijk and coworkers that explicitly take into account discrete blood vessels have been formulated in order to predict more accurately the overall temperature distribution inhomogeneity observed in clinical practice. This temperature inhomogeneity cannot be predicted with the more popular Pennes bioheat equation [45]. In Pennes equation, the influence of the blood flow is taken into account by a volumetric heat sink which assumes that the blood enters the local tissue volume at the arterial temperature (about 37°C in humans) and leaves the tissue at the local tissue temperature. However, the assumption that all the tissue-vascular heat exchange takes place in the capillary bed is not correct [7, 46, 47]. Chen and Holmes [42] and Chato [43] were the first to point out that the thermal equilibration process takes place not into the capillaries, as it was assumed by Pennes, but in vessels with a diameter between 0.2 and 0.5 mm. This fact has now been corroborated by several authors.

Other discrete blood vessels models have been proposed; see, for example, [47–51]. They mainly differ for the heat transfer equation in the tissue (in some models the Pennes' equation is used rather than (1)) and the way in which the vascular network is handled mathematically (with various degrees of complexity) in (2). The main challenge for the discrete vessel models is associated with solving (2) in the vessel-network domain. The difficulty is not only related to technical computer/software limitations but also with the availability of patient specific data. Although advancements in MRI/CT dynamic contrast imaging and MRI/CT angiography have started providing specific patient data about volumetric distribution of perfusion and morphology/flow rates of discrete vessels with increasing accuracy, they still cannot provide information for the thermally significant vessels in the diameter range 0.3–0.8 mm, required for a more accurate evaluation of the temperature increase [44].

Our discrete blood vessel model is based on the one described for scanned focused ultrasound hyperthermia by Lagendijk and coworkers [32] with several differences illustrated below. Contrary to [32], we have made no prior assumption about the heat transfer coefficient between the tissue and the blood vessels; the continuity condition applied at the boundary between the tissue domain and the vessel domain implicitly models this. This is especially important with pulsed focused ultrasound, where the Nusselt number (ratio between the heat transfer coefficient and the conductive heat transfer coefficient) is changing along the blood vessel and also as a function of time. Most of the discrete blood vessels models employ a Nusselt number constant along the vessel to describe the heat transfer between the flowing blood and the tissue. The advantage of assuming a constant Nusselt number is that it considerably simplifies the computational problem allowing the possibility of dealing with more complex vascular geometries than the one considered here.

At first, we have considered the situation in which a single blood vessel is directly targeted, that is, the ultrasound focus is at the vessel center and the vessel is along the axial direction of the ultrasound field (Figures 1–4). Kolios et al. [49] and Lagendijk et al. [32] have considered similar situations using a finite difference technique.

We have seen that the vessel size and blood flow strongly affect the temperature reached at the vessel wall with the largest vessels proving very difficult to heat and the smallest ones, less than 0.3 mm in radius, heating easily to the nearby tissue temperature. For sufficiently long insonation time, peak power, or ultrasound width and/or height, the temperature at the vessel wall can be significantly increased at the cost of producing nearby regions of thermal damage. When the focus is, for example, one mm from the vessel wall (Figure 5(a)), temperatures favorable to thermal damage can be easily reached at the focal plane, but the temperature distribution is highly inhomogeneous because of the blood vessel presence. This situation remains true even when the vessel makes an angle different from zero with the axial direction of the ultrasound field. Since several thermally significant vessels run in counterflow pairs, we have considered a few examples of a single vessel pair (Figure 6). The results are very similar to the ones obtained for a single vessel with even a larger temperature heterogeneity caused by the presence of two nearby blood vessels.

We have then simulated ultrasound treatments in a homogeneous block of muscle-like tissue containing a set of thermally significant blood vessel pairs and calculated the thermal dose associated with each treatment. In our plots, the thermal dose has been normalized with respect to the thermal dose of 240 equivalent minutes at 43°C. In normal thigh muscle in pig [38, 40], a dose threshold of 240 minutes at 43°C is considered adequate to coagulate all tissue, that is, correspond to severe damage with 100% necrosis. For minimal necrosis (partial damage) in normal thigh muscle, a thermal dose of 31.2 equivalent minutes at 43°C has been reported in rabbit thigh muscle [52]. Therefore, a thermal dose of 240 equivalent minutes at 43°C can be considered as a conservative estimate.

In general, studies have shown that that the threshold for thermal damage (necrosis) in different normal tissues in various species including humans ranges from 20 to 240 equivalent minutes at 43°C [38, 40]. Fewer studies have considered the threshold for thermal damage on tumor tissue (see e.g., [38, 53]). In [53], data from heating human breast carcinomas and surrounding normal tissue have been reported. The thermal dose required for inducing 50% of tissue necrosis was calculated to be  116 ± 31  equivalent minutes at 43°C for the malignant tissue and  205 ± 49  equivalent minutes at 43°C for normal tissue. Similarly, the thermal doses that caused damage to 50% of the vessels were found to be, respectively, 63 ± 34  and  144 ± 46  equivalent minutes at 43°C for the malignant and the normal vessels. Therefore, tissue and blood vessels in tumors seem to be more sensitive to heat than is the surrounding normal tissue and vessels.

In our simulations, we see that, when the treatments are delivered without taking into account the cooling effect exerted by the blood flow, the resulting thermal dose is highly variable with regions of thermal damage, regions of underdosage close to the vessels, and areas in between these two extremes. This is true for all the three delivery methods examined in this investigation. However, the random insonation treatment with fixed power has a much lower peak thermal dose, about 3 to 4 times smaller than the peak thermal dose obtained with the sequential or spiral delivery methods.

When the power was adjusted so as to have a thermal dose accumulation at each insonation close to the threshold for thermal damage, the resulting total thermal dose distribution is more uniformly distributed in all three delivery methods. Nevertheless, areas of thermal damage and underdosage are still present. The average peak power and standard deviation used in the treatments are, respectively, for case (i) 18.4 ± 3.8 W/cm^3^, for case (ii) 17.1 ± 3.9 W/cm^3^, and for case (iii) 16.5 ± 4.1 W/cm^3^. Therefore, the random treatment requires, on average, more power than the other two delivery methods.

In principle, one could adjust the power at each insonation so as to have peak values for the total thermal dose below or close to one; however, this would be at the risk of producing regions of underdosage. Since the power required to keep the thermal dose close to the threshold for thermal damage at each insonation varies greatly from one insonation to next even for nearby points, only a treatment planning able to predict the overall temperature distribution as a function of acoustic energy absorption, blood vessel position, and blood flow would be able to produce the required thermal dose distribution. Such a treatment planning would require the exact perfusion and the location of the thermally significant intermediate blood vessels (0.3–0.8 mm) of each individual patient. This information is currently not available with the required resolution. To compensate for this missing information, a thermal feedback control strategy could be implemented. One possible strategy would be to perform a treatment of type (i) random pattern treatment on a given grid and after a given amount of time to measure the temperature using MRI thermometry. Regions whose temperature rise is below a given set threshold could undergo further treatment. This and other thermal feedback control methods will greatly benefit from an improvement of the current resolution of MRI thermometry, which is of the order of one mm while temperature heterogeneity is expected to be present at a level of about 0.3 mm.

It is expected that a thermal feedback control strategy would significantly increase the duration of a treatment since regions of underdosage would require a further treatment. This shortcoming could be compensated with technological advances able to speed up the movement and the change of orientation of the transducer during the treatment.

## 5. Conclusions

Several challenges need to be overcome in order to develop a sound thermal treatment planning for focused ultrasound. Even in the simplified situation discussed in this paper, we have seen a wide variability in temperature/thermal dose distribution achieved, with regions of both underdosage around the vessels and thermal damage in the same treatment.

The temperature increase in the tissue is determined by the amount of ultrasound energy deposited and the amount of tissue cooling by thermal conductance and blood flow convection. No matter which delivery method is chosen, the only way to control temperature is to adjust the energy deposition to compensate for the presence of thermally significant vessels in the target area. By doing this, we have shown that it is possible to reduce the temperature heterogeneity observed in focused ultrasound thermal treatments.
